# Pregnancy-Associated Plasma Protein-A and Free β-Human Chorionic Gonadotrophin in Relation with Oxidative Stress in Obese Pregnant Women: A Clinical Cross-Sectional Study

**DOI:** 10.3390/pathophysiology31030037

**Published:** 2024-09-19

**Authors:** Vanja Dimitrov, Maria Mikerova, Vladimir Reshetnikov, Victor Mikhailovsky, Sasa Raicevic, Sergey Bolevich, Vladimir Jakovljevic, Tamara Nikolic Turnic

**Affiliations:** 1 Clinic for Obstetrics and Gynecology, University Clinical Center Nis, 18000 Nis, Serbia; vanjadimitrov@gmail.com; 2 N.A. Semashko Public Health and Healthcare Department, F.F. Erismann Institute of Public Health, I.M. Sechenov First Moscow State, 119435 Moscow, Russia; mikerova_m_s@staff.sechenov.ru (M.M.); resh1960@mail.ru (V.R.); mikhaylovskiy_v_v@staff.sechenov.ru (V.M.); 3 Department of Gynecology and Obstetrics, Medical Faculty, University of Montenegro, 81000 Podgorica, Montenegro; sasar@t-com.me; 4 Department of Human Pathology, 1st Moscow State Medical, University IM Sechenov, Trubetskaya Street 8 St., 119991 Moscow, Russia; bolevich2011@yandex.ru (S.B.); drvladakgbg@yahoo.com (V.J.); 5 Department of Physiology, Faculty of Medical Sciences, University of Kragujevac, Svetozara Markovica 69, 34000 Kragujevac, Serbia; 6 Center of Excellence for Redox Balance Research in Cardiovascular and Metabolic Disorders, 34000 Kragujevac, Serbia; 7 Department of Pharmacy, Faculty of Medical Sciences, University of Kragujevac, Svetozara Markovica 69, 34000 Kragujevac, Serbia

**Keywords:** oxidative stress, obesity, early pregnancy, prediction

## Abstract

**Background**: The pathophysiological mechanism underlying pregnancy complications is not entirely known. Although it is currently impossible to predict the occurrence of redox imbalance, it is possible to identify women with a high or medium risk of developing this disease prior to a negative outcome by non-invasive diagnostic methods. **The Aim**: This study aimed to examine the possible role of the parameter of oxidative stress (OS) measured in early pregnancy in the screening/treatment of obesity and its complications during pregnancy. **Methods**: This research was designed as a prospective observational cross-sectional clinical study which included 40 non-obese and 31 obese pregnant women between 11 and 13 g.w. who were managed in the Department of Obstetrics, University Clinical Center Kragujevac in Serbia. We collected anthropometric and clinical indicators, maternal and pregnancy factors, and measured prooxidative parameters from blood samples. **Results**: We observed significantly increased levels of the superoxide anion radical, hydrogen peroxide and the index of lipid peroxidation in the Obese group in comparison with the Non-Obese group and significantly decreased bioavailability of nitrites in the Obese group in comparison with the Non-Obese group. **Conclusions**: The determination of systemic parameters of OS in early pregnancy could be a good methodological approach in the screening/treatment of obesity during pregnancy and this approach should be followed for the screening of endothelial dysfunction in pregnancy which needs further monitoring and/or treatment.

## 1. Introduction

The term oxidative stress (OS) is related to the state of imbalance between the production of reactive oxygen species (ROS) and the power of antioxidant mechanisms to reduce them. This can be a result of an increase in ROS generation or a weakening of antioxidant defense [1]. During the analysis of the previous literature data, we have found that the topic of OS in pregnancy is becoming more common, and knowledge of this theme is increasing. Elevated OS has adverse effects on pregnancy, pregnant health, and especially on fetal growth, and could be a reason for the incorrect implantation of embryos, miscarriages, premature and low births, as well as malformations [2].

A very serious disorder that is associated with endothelial dysfunction and elevated OS with impaired circulation in the placenta is preeclampsia (PE) and eclampsia [1,2,3]. PE is a multisystem progressive disease that specifically occurs in the population of older pregnant women (above 35 years old) who are in the third trimester. This disease is manifested by new-onset hypertension and proteinuria, with or without edema, which can progress to the dysfunction of various organ systems such as the cerebral system, liver, kidneys, and many other organs [1,2,3]. PE carries with it significant risks of fetal and neonatal complications, as well as complications for the mother’s health [4].

OS is one of the states that directly contribute to the development of PE. It is extremely significant to recognize pregnant women with an increased risk since they require more intensive monitoring, and in some cases, require preventive measures [5,6,7]. Other risk factors are genetic factors, especially the polymorphism of many genes, inflammatory conditions, thrombophilia, and increased blood pressure [8,9,10]. OS is an imbalance of redox status (high concentrations of prooxidants and low activity of the antioxidant system) in the body and a very important factor for the development of diseases or disorders [11,12]. Also, pregnancy is well known to increase OS, a phenomenon generated by a normal systemic inflammatory response, which results in high amounts of circulating ROS. The major source of ROS during pregnancy is the placenta, as the central organ that regulates redox balance.

In the main pregnancy organ placenta, OS and the high metabolism of nutritive materials are common. Sometimes, ROS harm placental development and regulate gene transcription and downstream activities, such as trophoblast proliferation and growth [13,14]. Previous studies have reported a link between OS and pregnancy complications that in many cases hinder fetal development [15,16,17,18]. It is known that the major cause of pregnancy complications lies in a lack of oxygen delivery and nutrition, which leads to hypoplasia and disrupted placental function [15,16,17,18]. These premature changes, if they occur, could be the reason for placental injury and first-trimester spontaneous abortions [15,16,17,18]. If the pregnancy goes on and is stable, insufficient placental perfusion and ischemia/reperfusion (I/R) induces OS, which is directly associated with intrauterine growth restriction (IUGR) [16,17].

Actually, the placenta is an intermediary organ that balances chole metabolism between the mother and the fetus and plays a critical role in modulating the intrauterine environment and fetal growth. Since obesity is a maternal risk factor for cardiovascular disorders and IUGR, we know that obesity is linked with inflammation of the placenta, placental dysfunction, and probably OS [17].

Changes in nitric oxide (NO) and ROS are also seen in pathological pregnancies, such as in PE [16,17]. ROS stimulate platelet adhesion and aggregation, which impairs uteroplacental blood flow and may cause placental infarction. As a result, the amount of oxygen and nutrients necessary for the proper development of the fetus decreases [16,17,18].

On the other hand, the main perinatal parameters that represent the clinical picture of pregnancy are PAPP-A (Pregnancy Associated Plasma Protein A) and ß-hCG (beta-Human Chorionic Gonadotropin).

Based on the known data reference range of the PAPP-A and Beta-hCG, these are divided into the levels normal (0.5–2.5 MoM) or abnormal (low: <0.49 MoM and high: >2.5 MoM), and could be a sign of pregnancy complications and fetal impairments [19]. The values of PAPP-A < 0.5 MoM at 11–13 gestational weeks of pregnancy could be predictive values for premature births of the low weight births and/or low fetal cranial circumference [19].

On the other hand, higher β-hCG levels are associated with improved clinical outcomes and live births. Still, there is no literature data that have investigated the connection of these markers with obesity and redox status in pregnancy [20,21,22].

The pathophysiological mechanism underlying pregnancy complications is not entirely known. The main question is which course will OS take during pregnancy and whether it is linked to some disorder, for example, obesity. Although it is currently impossible to predict the occurrence of redox imbalance and the consequences of obesity and its serious complications—including preeclampsia as a major generator of oxidative overproduction—it is possible to identify women with a high or medium risk of developing redox imbalance and placental dysfunction prior to a negative outcome by non-invasive diagnostic methods. There is no actual screening test for placental dysfunction that includes any measured biochemical markers, and all screening methods are based on anamnestic data and ultrasound examination during pregnancy.

The significance of this article lies in the creation of a good methodological approach in the screening/treatment of obesity in pregnant women who are likely to develop significant redox imbalance and need further monitoring and/or treatment. Also, this study aimed to find a potential association between β-hCG and PAPP-A, obesity, and oxidative stress in pregnant women.

## 2. Material and Methods

### 2.1. Ethical Concerns

This research was conducted in accordance with the Good Clinical Practice and Helsinki Declarations (revision 2013). Also, this study was approved by the Ethics Committee of the University Clinical Center in Kragujevac, Serbia (number of approval 01/23-128 from date 10 February 2023). Prior to inclusion in the study, we obtained voluntary written and informed patient consent.

### 2.2. Design of Study and Participants

This research was designed as a prospective observational cross-sectional clinical study that included 71 pregnant women (40 non-obese and 31 obese women) between 11 and 13 gestational weeks who were managed in the Department of Obstetrics, University Clinical Center Kragujevac in Serbia between 1 March and 30 June 2023. The University Clinical Center Kragujevac in Serbia is a tertiary institution in the central part of Serbia which provides specialist healthcare for a small number of pregnant women with a pathological pregnancy. The main inclusion criteria were singleton pregnancies, the absence of serious diseases (a disease that must include therapy or the cessation of pregnancy), or malignancies, while the exclusion criteria were pregnancy with major fetal abnormalities, bleeding in pregnancy, and a negative outcome before 24 gestational week (miscarriage).

### 2.3. Anamnestic Data

Between 11 and 13 gestational weeks, at the first examination, we collected the following data from pregnant women: basic demographic and socio-epidemiological data such as age, present comorbidities (hypertension, diabetes, thyroid disorders, antiphospholipid syndrome, and/or thrombophilia), previous pregnancies and births, cigarette and alcohol consumption, the method of conception, medicamentous anamnesis (the use of antihypertensives, antidepressants, antiepileptics, aspirin, corticosteroids, insulin, and/or thyroxine), parity, previous pregnancies with complications, positive family history for pregnancy complications, and additional pregnancy-related data such as the gestational week and the time interval from previous pregnancies.

### 2.4. Anthropometric Measures

The body mass index (BMI) was used as a marker of obesity according to the following categories for women: less than <24.9, observed within the healthy weight range (Non-Obese group), and from 25.0 to <29.9 and 30 or higher, observed as overweight and in the obesity range (Obese group).

### 2.5. Maternal and Pregnancy Factors

At the first examination and after inclusion in the study, clinical examination determined the following parameters: ultrasound confirmation of gestational age, the body weight of the pregnant woman, body height, the BMI, systolic and diastolic blood pressure, presence of proteinuria, as well as serum levels of β-hCG, PAPP-A, fetal CRL (crown–rump length), and NT (nuchal translucency) among pregnant women. Obtaining the results of these measurements did not affect the further course and management of the pregnancy.

β-hCG and PAPP-A were determined at 11–13 weeks of gestation and from the serum sample from all patients in the referent biochemical laboratory of the Clinical Center Kragujevac, Serbia. Blood samples were collected by a healthcare worker by venous punction of the cubital vein for serum separating. Blood samples were further processed according to the standardized biochemical procedures.

### 2.6. Determination of β-hCG and PAPP-A Markers

β-hCG was determined using the ELISA method, an enzyme immunoassay for quantitative in vitro diagnostic analysis in serum. We used a β-hCG ELISA kit (Sigma-Aldrich, St. Louis, MO, USA) based on the sandwich principle. The procedure is based on coated wells with monoclonal antibodies. After the incubation period, the unbound conjugate was washed off and the total amount of β-hCG was proportional to the concentration of β-hCG/hCG in the serum sample.

The PAPP-A marker was determined by using DELFIA^®^ and AutoDELFIA^®^ PAPP-A kits (Revvity, Waltham, MA, USA) for the quantitative determination of this marker from maternal serum. This procedure is based on solid-phase, two-site fluoroimmunometric assays with the indirect sandwich method. These biochemical procedures were performed in the reference biochemical laboratory of the Clinical Center Kragujevac in Kragujevac, Serbia.

### 2.7. Maternal Blood Sample Preparation and Determination of OS Markers

From all participants at 11–13 gestational weeks, blood samples were collected into EDTA tubes for plasma and lysate collection. From the blood samples, red blood cells were separated by centrifugation at 2500× *g* at 4 °C for 5 min, and then the plasma and erythrocyte samples were stored at −80 °C until use.

The determination of the biomarkers of oxidative stress was performed in the Laboratory of Cardiovascular Physiology at the Faculty of Medical Sciences, University of Kragujevac. We applied spectrophotometric methods using Shimadzu UV 1800, Kyoto, Japan for the plasma samples. We measured the index of lipid peroxidation (TBARS), nitrites (NO_2_^−^), superoxide anion radical (O_2_^−^), and hydrogen peroxide (H_2_O_2_).

Superoxide anion radical determination was performed using on a nitroblue tetrazolium reaction with an assay mixture and a measuring wavelength of 530 nm [23]. Hydrogen peroxide determination was performed through the horseradish peroxidase oxidation of phenol red and a measuring wavelength of 610 nm [24]. Nitric oxide was measured indirectly by measuring the nitrites in the plasma samples. This method was based on the reaction of Griess’ reagent and sodium nitrite and a measuring wavelength of 543 nm [25]. The index of lipid peroxidation was determined using 1% thiobarbituric acid in 0.05 of sodium hydroxide and a measuring wavelength of 530 nm [26]. For all analyses, distilled water was used as a blank control.

### 2.8. Sample Size Calculation and Statistical Data Analysis

The calculation of the total sample was based on the results of the previously published Cruickshank T study [27], in which the predictive value of factor 15 for PE was estimated. For the calculation, a *t*-test for the paired sample was used which was two-tailed, assuming an alpha error of 0.05 and a study power of 0.8. (beta bug 0.2), the using G-Power softwar for 3.1. 9.6 for Mac OS. Taking into account the results of the aforementioned study, the total number of participants was calculated as 80 in both groups (1:1 ratio); 9 participants were excluded from the analysis due to incomplete data (11.3%).

All data are presented as the mean ± standard deviations or as the frequency in percent (%). Statistical significance was estimated using a Chi-square test with statistical significance at a level of *p* < 0.05. A Chi-squared test or a Student *t*-test was used to test the differences between the selected variables/parameters between the groups with a significance level of 0.05. Pearson’s correlation and the linear regression model were used to test the significant association between the variables. All analyses were performed in a licensed statistical program, SPSS version 26 (IBM SPSS for Macintosh). Graphs were created in the Microsoft Office Excell Program version 2023 for Mac OS 

## 3. Results

### 3.1. Basic Demographic and Anthropometric Data of Study Group

This study included 71 pregnant women with an age of 27.59 ± 3.85 years who were at 12.39 ± 0.64 gestational weeks. Furthermore, 100% of the participants were women who had a spontaneous conception with a mean time of 48.69 ± 21.11 months from a previous pregnancy, and about half of them were pregnant for the first time (50.70%). Also, all the baseline demographic data for both groups are shown in Table 1.

Table 2 presents the anamnestic data of the study population according to the BMI category. Most of the pregnant women did not have any present comorbidity. Also, none of the listed drugs were used among the study groups (Table 2).

### 3.2. Fetal Markers

Fetal parameters such as CRL, NT, B-hCG, and PAPP-A are presented in Table 3. All the parameters were in a referent range according to their gestational week (Table 3).

#### Pro-Oxidative Blood Markers in Early Pregnancy

**Figure 1:**
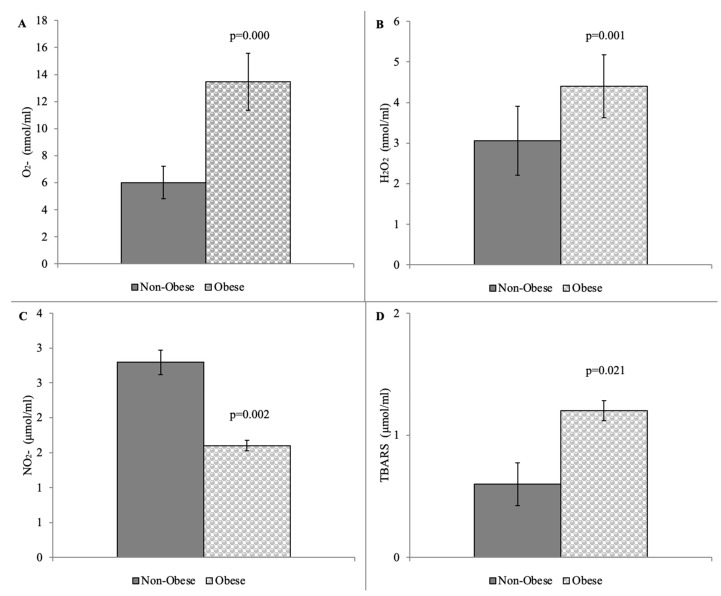
Prooxidative parameters among the Non-Obese and Obese group: (**A**) superoxide anion radical, O_2_^−^ (**B**) hydrogen peroxide, H_2_O_2_; (**C**) nitrites, NO_2_^−^ (**D**) index of lipid peroxidation, TBARS. The results are presented as the mean and SD. Statistical significance was confirmed by the Student *t*-test.

The results of the prooxidative markers are shown in Figure 1. We observed significantly increased levels of the superoxide anion radical, hydrogen peroxide, and the index of lipid peroxidation in the Obese group in comparison with the Non-Obese group (Figure 1A,B,D) and significantly decreased bioavailability of nitrites in the Obese group in comparison with the Non-Obese group (Figure 1C).

### 3.3. Correlation Analysis of Prooxidant Markers and Fetal and Maternal Markers

The results of the correlation analysis are shown in Table 4. We observed a statistically significant positive correlation between the superoxide anion radical, hydrogen peroxide, and the index of lipid peroxidation with body weight, the BMI, and PAPP-A. Also, this test confirmed an inverse correlation of the superoxide anion radical, hydrogen peroxide, and the index of lipid peroxidation with the levels of B-hCG from the blood samples. In addition, the nitrites showed an inverse correlation with body weight and the BMI, and a linear positive relation with B-hCG and PAPP-A (Table 4). These results confirmed the significance of the determination of concentrations of prooxidative markers in pregnant women in early pregnancy, and we can conclude that obesity and pregnancy together could be a generator of the overproduction of OS (Table 4). Regarding the correlation analysis of the perinatal factors between each other, we can observe that all of them were connected and most correlated in positive manner, with the exception of the PAPP-A which had an inverse correlation with other parameters (Table 5).

## 4. Discussion

Our research was conducted prospectively and included obese and non-obese pregnant women with an average age of 27.59 ± 3.85 years and 12.39 ± 0.64 gestational weeks who were managed at the Department of Obstetrics, University Clinical Center Kragujevac in Serbia between 1 March and 30 June 2023. This study aimed to examine the possible role of the parameter of OS measured in early pregnancy in the screening of obesity and its potential oxidative complications during pregnancy, such as hypertension, diabetes, PE, and HELLP syndrome.

OS is the main pathophysiological mechanism in many conditions, and decreasing it could prevent many diseases [6,7,27,28]. During obesity in pregnancy, the overproduction of OS is present, which contributes to the structural damage of cells and to the development of pregnancy-related complications. The main sources of OS in these states are vascular components, such as the placenta, which requires OS for vascular formation [29]. As a consequence, some very serious vascular disorders, such as preeclampsia, eclampsia, diabetes, thrombophilia, etc., can be expected [29]. PE affects 5–10% of all pregnant women and is one of the leading causes of maternal death, admission to intensive care, cesarean section, and premature pregnancies [30,31]. Therefore, it is very important to observe all of the known risk factors in a pregnant woman to recognize the clinical manifestations of endothelial dysfunction and prevent possible complications that may arise during pregnancy or after childbirth.

Our study noticed that an increased maternal BMI was associated with higher levels of oxidative stress (Figure 1). As for obesity, certain mechanisms have been described that explain its role in the development of placental dysfunction. In comparison with normal weight, obesity induces inflammatory damage in adipose tissue and in that way, increases the risk for PE [32,33,34,35]. With the prevalence of maternal obesity and the increase in PE cases worldwide, it is necessary to examine the impact of obesity on both the course and outcomes of PE. Taking into account adipose tissue, which is composed of adipocytes and immune and vascular cells, this tissue is an endocrine organ that participates in the modulation of various immune responses, and thus the impact of its pathophysiological mechanisms on PE has been assumed [36,37,38]. As we know, adipose tissue produces adipokines such as TNF-α and IL-6 that can further promote the cell-to-cell signaling of local and systemic inflammation. In obese women during pregnancy, maternal adipose tissue contributes to reduced placental vascularization during proinflammatory states and a huge immune response. In that sense, such a release of proinflammatory cytokines and other factors promotes ischemia in the placenta, which leads to maternal hypertension and a reduction in fetal growth [39].

The meta-analysis of Moteza et al. [39] analyzed the relationship between the BMI and PE among 5946 samples. They confirmed that there is a significant relationship between the BMI, OS, and the risk of PE, so it can be said that the BMI may be one of the ways to diagnose serious complications such as PE. When assessing the importance of pregnancy complication risk factors, it is necessary to take into account that a common factor is obesity. Also, a meta-analysis showed that elevated levels of total cholesterol and triglycerides during pregnancy were significantly associated with the risk of PE [40]. Cholesterol is necessary for the synthesis of placental steroids, and an increase in cholesterol levels during pregnancy promotes the accumulation of maternal fat stores in the first two trimesters of pregnancy. In clinical practice, it is very useful to monitor these parameters because of their important role in many disorders.

On the other hand, we analyzed the values of fetal markers in relation to other variables. A trophoblast invasion disorder is cited as a reason for the reduced production of PAPP-A [38,41]. A level equal to or greater than 0.5 is considered normal, while levels less than 0.5 are labeled as low. In our study, the average value of this protein was 3.07, with the lowest recorded value being 0.27 and the highest being 112.0. The PAPP-A marker was significantly higher in the Obese group of participants in comparison with the Non-Obese group (Table 3).

So far, it has been shown that PE is associated with a disturbance in PAPP-A levels, and what is significant is that the highest values were recorded before signs of the disease were evident [41]. Certain authors described no difference in PAPP-A levels in patients suffering from mild PE (hypertension in late pregnancy without albuminuria) but noted a significant difference in values in severe PE (with albuminuria) compared with normal values for the corresponding stage of gestation [37,38,41]. Narges Moslemi et al. examined the role of PAPP-A in the prediction of PE. They concluded that it is possible to benefit from the measuring of PAPP-A in the first and second trimesters, especially their cumulative values in both trimesters, for the prediction of the incidence of PE with high specificity and sensitivity [41]. Another study also confirmed the role of PAPP-A: very low levels during the first trimester are very often associated with the development of preeclampsia later in pregnancy [42].

Apart from the routine measurement of this parameter in the first trimester to determine chromosomal abnormalities, it is considered that PAPP-A measured between 11 and 13 gestational weeks can effectively be used early in combination with a Doppler ultrasound and possibly other biochemical markers and maternal factors for PE screening [41]. In a previous prospective cohort study, it was shown that PAPP-A negatively correlated with the uterine artery pulsatility index [42,43,44]. In our study, a statistically significant linear correlation was observed between PAPP-A and prooxidant markers, as well as between β-hCG and all tested prooxidative parameters. Definitely, one of the very important markers in the screening of PE is the determination of the values of PAPP-A, PGIF, and BMI in 10–13 g.w., also called screening for PE. The results of our study are in accordance with the literature data and confirm that the PAPP-A marker could be a predictor of obesity and elevated OS in the second and third trimester of pregnancy [44].

Another very important fetal factor is β-hCG, which was positively associated with prooxidants and the BMI in our study (Table 3 and Table 4). Also, the correlation analysis showed that the BMI was positively correlated with β-hCG. A moderate inverse correlation was present in the connection between the BMI and β-hCG (Table 4). The very important result of this is that the PAPP-a marker was associated with several variables and showed a negative strong correlation with other fetal markers, such as CRL and NT (Table 4).

The limitation of this study at this moment lies in the absence of postpartum maternal and neonatal characteristics of the participants, where we could correlate them with the data from the beginning of the pregnancy. This study should also have a broader methodological approach in order to assess the clinical significance of OS markers. Since this study is still ongoing, we could provide these data at the end of each pregnancy of our participants when we obtain additional information in the future.

## 5. Conclusions

The determination of systemic parameters of OS in early pregnancy could be a good methodological approach in the screening/treatment of obesity during pregnancy. OS could be directly linked with many fetus conditions and fetal development in utero. This approach should be followed for the screening of endothelial dysfunction in pregnancy which needs further monitoring and/or treatment.

## Figures and Tables

**Table 1:** Basic demographic and anthropometric characteristics of both groups (Non-Obese and Obese). The results are presented as the mean and SD or as the frequency in percent (%). Statistical significance was confirmed by the Student *t*-test (continuous) or Chi-squared test (categorical).

Variables	Non-Obese Group (Mean ± SD) (n = 40)	Obese Group (Mean ± SD) (n = 31)	*p*
Age old [years]	29.92 ± 4.37	27.13 ± 3.01	0.679
Body weight [kg]	69.02 ± 2.21	97.09 ± 2.11	0.000
Body height [cm]	167.33 ± 8.19	167.58 ± 5.07	0.770
Body mass index [kg/m^2^]	21.08 ± 2.27	27.91 ± 4.04	0.000
Time from previous pregnancy [months]	36.88 ± 4.18	60.81 ± 1.34	0.000
Gestational week	12.42 ± 0.71	12.35 ± 0.55	0.998
First pregnancy [%]	Yes 16 [40%]	Yes 20 [64.51%]	0.233
Spontaneous pregnancy conception [%]	Yes 40 [100%]	Yes 31 [100%]	0.999

**Table 2:** Anamnestic data of both groups. The results are presented as the frequency in percent (%).

Variables	Non-Obese Group (Mean ± SD) (n = 40)		Obese Group (Mean ± SD) (n = 31)		*p*
Positive hereditary PE [%]	Yes 0 [0%]	No 40 [100%]	Yes 0 [0%]	No 31 [100%]	0.889
Smoking before pregnancy [%]	Yes 0 [0%]	No 40 [100%]	Yes 0 [0%]	No 31 [100%]	0.890
Smoking during pregnancy [%]	Yes 0 [0%]	No 40 [100%]	Yes 0 [0%]	No 31 [100%]	0.923
Alcohol consumption before pregnancy [%]	Yes 0 [0%]	No 40 [100%]	Yes 0 [0%]	No 31 [100%]	0.11
Alcohol consumption during pregnancy [%]	Yes 0 [0%]	No 40 [100%]	Yes 0 [0%]	No 31 [100%]	0.937
Hypertension [%]	Yes 0 [0%]	No 40 [100%]	Yes 0 [0%]	No 31 [100%]	0.899
Diabetes Mellitus [%]	Yes 0 [0%]	No 40 [100%]	Yes 1 [3.2%]	No 30 [96.8%]	0.602
Antiphospholipid syndrome [%]	Yes 0 [0%]	No 40 [100%]	Yes 0 [0%]	No 31 [100%]	0.902
Thrombophilia [%]	Yes 0 [0%]	No 40 [100%]	Yes 0 [0%]	No 31 [100%]	0.877
Antihypertensives before pregnancy [%]	Yes 0 [0%]	No 40 [100%]	Yes 0 [0%]	No 31 [100%]	0.901
Antihypertensives during pregnancy [%]	Yes 0 [0%]	No 40 [100%]	Yes 0 [0%]	No 31 [100%]	0.967
Antidepressant before pregnancy [%]	Yes 0 [0%]	No 40 [100%]	Yes 0 [0%]	No 31 [100%]	0.988
Antidepressant during pregnancy [%]	Yes 0 [0%]	No 40 [100%]	Yes 0 [0%]	No 31 [100%]	0.956
Antiepileptics before pregnancy [%]	Yes 0 [0%]	No 40 [100%]	Yes 0 [0%]	No 31 [100%]	0.978
Antiepileptics during pregnancy [%]	Yes 0 [0%]	No 40 [100%]	Yes 0 [0%]	No 31 [100%]	0.989
Aspirin before pregnancy [%]	Yes 0 [0%]	No 40 [100%]	Yes 0 [0%]	No 31 [100%]	0.899
Aspirin during pregnancy [%]	Yes 0 [0%]	No 40 [100%]	Yes 0 [0%]	No 31 [100%]	0.923
Corticosteroids before pregnancy [%]	Yes 0 [0%]	No 40 [100%]	Yes 0 [0%]	No 31 [100%]	0.945
Corticosteroids during pregnancy [%]	Yes 0 [0%]	No 40 [100%]	Yes 0 [0%]	No 31 [100%]	0.998
Insulin before pregnancy [%]	Yes 0 [0%]	No 40 [100%]	Yes 1 [3.2%]	No 30 [96.8%]	0.623
Insulin during pregnancy [%]	Yes 0 [0%]	No 40 [100%]	Yes 1 [3.2%]	No 30 [96.8%]	0.713
Thyroxine before pregnancy [%]	Yes 0 [0%]	No 40 [100%]	Yes 0 [0%]	No 31 [100%]	0.988
Thyroxine during pregnancy [%]	Yes 0 [0%]	No 40 [100%]	Yes 0 [0%]	No 31 [100%]	0.967

**Table 3:** Fetal parameters among both groups (Non-Obese and Obese). The results are presented as the mean and SD or as frequency in percent (%). Statistical significance was confirmed by the Student *t*-test.

Variables	Non-Obese Group (Mean ± SD) (n = 40)	Obese Group (Mean ± SD) (n = 31)	*p*
Fetal crown–rump length (CRL) [cm]	61.54 ± 5.93	61.83 ± 13.75	0.886
Fetal nuchal translucency (NT) [mm]	1.49 ± 0.26	1.40 ± 0.25	0.651
Free β-human chorionic gonadotrophin (B-hCG) [mUI/mL]	1.59 ± 0.83	1.11 ± 0.68	0.000
Pregnancy-associated Plasma Protein A (PAPP-A) [MOM]	1.50 ± 0.85	4.98 ± 0.99	0.000

**Table 4:** Correlation analysis between perinatal parameters and OS markers among the study population. The results are presented as r (correlation coefficient) and *p* (statistical significance). Statistical significance was confirmed by Person’s correlation analysis.

Variables	Superoxide Anion Radical [nmol/mL]	Hydrogen Peroxide [nmol/mL]	Nitrites [nmol/mL]	Index of Lipid Peroxidation [micromol/mL]
Age [years]	r = 0.667	r = 0.998	r = 0.994	r = 0.979
	*p* = 0.089	*p* = 0.243	*p* = 0.331	*p* = 0.353
Body weight [kg]	**r = 0.554**	**r = 0.452**	**r = −0.199**	**r = 0.678**
	***p* = 0.000**	***p* = 0.000**	***p* = 0.000**	***p* = 0.000**
Body mass index [kg/m^2^]	**r = 0.567**	**r = 0.670**	**r = −0.223**	**r = 0.450**
	***p* = 0.000**	***p* = 0.000**	***p* = 0.000**	***p* = 0.000**
Time from previous pregnancy [months]	r = 0.442	r = 0.409	r = 0.471	r = 0.229
	*p* = 0.118	*p* = 0.134	*p* = 0.156	*p* = 0.191
Fetal crown–rump length (CRL) [cm]	r = 0.778	r = 0.765	r = 0.445	r = 0.405
	*p* = 0.243	*p* = 0.190	*p* = 0.221	*p* = 0.198
Fetal nuchal translucency (NT) [mm]	r = 0.677	r = 0.655	r = 0.509	r = 0.407
	*p* = 0.201	*p* = 0.199	*p* = 0.276	*p* = 0.202
Free β-human chorionic gonadotrophin (B-hCG) [mUI/mL]	**r = −0.134**	**r = −0.343**	**r = 0.302**	**r = −0.242**
	***p* = 0.021**	***p* = 0.033**	***p* = 0.019**	***p* = 0.022**
Pregnancy assocaited Plasma Protein A (PAPP-A) [MOM]	**r = 0.498**	**r = 0.245**	**r = 0.433**	**r = 0.331**
	***p* = 0.002**	***p* = 0.003**	***p* = 0.006**	***p* = 0.003**

Bold letters and numbers represent statistically significant values.

**Table 5:** Correlation analysis between perinatal parameters among the study population. The results are presented as r (correlation coefficient) and *p* (statistical significance). Statistical significance was confirmed by Pearson’s correlation analysis. Fetal crown–rump length (CRL) [cm], fetal nuchal translucency (NT) [mm], free β-human chorionic gonadotrophin (B-hCG) [mUI/mL], pregnancy-associated Plasma Protein-A (PAPP-A) [MOM].

Variables	CRL [cm]	NT [mm]	B-hCG [mUI/mL]	PAPP-A [MOM]
Fetal crown–rump length (CRL) [cm]	1	r = 0.234	**r = 0.378**	**r = −0.768**
		*p* = 0.002	***p* = 0.004**	***p* = 0.001**
Fetal nuchal translucency (NT) [mm]	**r = 0.234**	1	**r = 0.443**	**r = −0.356**
	***p* = 0.002**		***p* = 0.004**	***p* = 0.004**
Free β-human chorionic gonadotrophin (B-hCG) [mUI/mL]	**r = 0.378**	**r = 0.443**	1	**r = −0.242**
	***p* = 0.004**	***p* = 0.004**		***p* = 0.022**
Pregnancy-associated Plasma Protein-A (PAPP-A) [MOM]	**r = −0.768**	**r = −0.356**	**r = 0.433**	1
	***p* = 0.001**	***p* = 0.004**	***p* = 0.006**	

Bold letters represent statistically significant values.

## Data Availability

The data are available from the corresponding authors upon reasonable request.

## References

[1] Toboła-Wróbel K., Pietryga M., Dydowicz P., Napierała M., Brązert J., Florek E. (2020). Association of Oxidative Stress on Pregnancy. Oxidative Med. Cell. Longev..

[2] Aouache R., Biquard L., Vaiman D., Miralles F. (2018). Oxidative Stress in Preeclampsia and Placental Diseases. Int. J. Mol. Sci..

[3] Ghulmiyyah L., Sibai B. (2012). Maternal mortality from preeclampsia/eclampsia. Semin. Perinatol..

[4] Zhang J., Meikle S., Trumble A. (2003). Severe maternal morbidity associated with hypertensive disorders in pregnancy in the United States. Hypertens. Pregnancy.

[5] Duhig K., Chappell L.C., Shennan A.H. (2016). Oxidative stress in pregnancy and reproduction. Obstet. Med..

[6] Leslie K., Thilaganathan B., Papageorghiou A. (2011). Early prediction and prevention of pre-eclampsia. Best Pract. Res. Clin. Obstet. Gynaecol..

[7] Grzeszczak K., Łanocha-Arendarczyk N., Malinowski W., Ziętek P., Kosik-Bogacka D. (2023). Oxidative Stress in Pregnancy. Biomolecules.

[8] Hussain T., Murtaza G., Metwally E., Kalhoro D.H., Kalhoro M.S., Rahu B.A., Sahito R.G.A., Yin Y., Yang H., Chughtai M.I. (2021). The Role of Oxidative Stress and Antioxidant Balance in Pregnancy. Mediat. Inflamm..

[9] Mukherjee I., Dhar R., Singh S., Sharma J.B., Nag T.C., Mridha A.R., Jaiswal P., Biswas S., Karmakar S. (2021). Oxidative stress-induced impairment of trophoblast function causes preeclampsia through the unfolded protein response pathway. Sci. Rep..

[10] Mütze S., Rudnik-Schöneborn S., Zerres K., Rath W. (2008). Genes and the preeclampsia syndrome. J. Perinat. Med..

[11] Zhang C.X.W., Candia A.A., Sferruzzi-Perri A.N. (2024). Placental inflammation, oxidative stress, and fetal outcomes in maternal obesity. Trends Endocrinol. Metab..

[12] Mannino A., Sarapis K., Moschonis G. (2022). The Effect of Maternal Overweight and Obesity Pre-Pregnancy and during Childhood in the Development of Obesity in Children and Adolescents: A Systematic Literature Review. Nutrients.

[13] Weng J., Couture C., Girard S. (2023). Innate and Adaptive Immune Systems in Physiological and Pathological Pregnancy. Biology.

[14] Brouwers L., Franx A., Vogelvang T.E., Houben M.L., van Rijn B.B., Nikkels P.G. (2019). Association of Maternal Prepregnancy Body Mass Index with Placental Histopathological Characteristics in Uncomplicated Term Pregnancies. Pediatr. Dev. Pathol..

[15] Layden A.J., Bertolet M., Parks W.T., Adibi J.J., Roberts J.M., Catov J.M. (2023). Prepregnancy obesity and risk of placental inflammation at term: A selection bias analysis. Ann. Epidemiol..

[16] Musa E., Salazar-Petres E., Arowolo A., Levitt N., Matjila M., Sferruzzi-Perri A.N. (2023). Obesity and gestational diabetes independently and collectively induce specific effects on placental structure, inflammation and endocrine function in a cohort of South African women. J. Physiol..

[17] Sferruzzi-Perri A.N., Lopez-Tello J., Salazar-Petres E. (2023). Placental adaptations supporting fetal growth during normal and adverse gestational environments. Exp. Physiol..

[18] Napso T., Lean S.C., Lu M., Mort E.J., Desforges M., Moghimi A., Bartels B., El-Bacha T., Fowden A.L., Camm E.J. (2022). Diet-induced maternal obesity impacts feto-placental growth and induces sex-specific alterations in placental morphology, mitochondrial bioenergetics, dynamics, lipid metabolism and oxidative stress in mice. Acta Physiol..

[19] Patil M., Panchanadikar T.M., Wagh G. (2014). Variation of papp-a level in the first trimester of pregnancy and its clinical outcome. J. Obstet. Gynaecol. India.

[20] Lu J., Qi D., Xu W. (2022). Fertility-enhancing effect of oil-based contrast agents during hysterosalpingography and the variation of this effect within a 3-year follow-up period in infertile patients. Front. Med..

[21] Mannaerts D., Faes E., Gielis J., Van Craenenbroeck E., Cos P., Spaanderman M., Gyselaers W., Cornette J., Jacquemyn Y. (2018). Oxidative stress and endothelial function in normal pregnancy versus pre-eclampsia, a combined longitudinal and case control study. BMC Pregnancy Childbirth.

[22] https://isshp.org/isshp-guidelines-for-hypertensive-disorders-of-pregnancy-published/.

[23] Ohkawa H., Ohishi N., Yagi K. (1979). Assay for lipid peroxides in animal tissues by the thiobarbituric acid reaction. Anal. Biochem..

[24] Green L.C., Wagner D.A., Glogowski J., Skipper P.L., Wishnok J.S., Tannenbaum S.R. (1982). Analysis of nitrate, nitrite and [15 N] nitrate in biological fluids. Anal. Biochem..

[25] Auclair C., Voisin E., Greenvvald R.A. (1985). Nitroblue Tetrazolium Reductionin: Handbook of Methods for Oxygen Radical Research.

[26] Pick E., Keisari Y. (1980). A simple colorimetric method for the measurement of hydrogen peroxide produced by cells in culture. J. Immunol. Methods.

[27] Cruickshank T., MacDonald T.M., Walker S.P., Keenan E., Dane K., Middleton A., Kyritsis V., Myers J., Cluver C., Hastie R. (2021). Circulating growth differentiation factor 15 is increased preceding preeclampsia diagnosis: Implications as a disease biomarker. J. Am. Heart Assoc..

[28] Duckitt K., Harrington D. (2005). Risk factors for pre-eclampsia at antenatal booking: Systematic review of controlled studies. BMJ.

[29] Moghaddas Sani H., Zununi Vahed S., Ardalan M. (2019). Preeclampsia: A close look at renal dysfunction. Biomed. Pharmacother..

[30] Rana S., Lemoine E., Granger J.P., Karumanchi S.A. (2019). Preeclampsia: Pathophysiology, Challenges, and Perspectives. Circ. Res..

[31] Paré E., Parry S., McElrath T.F., Pucci D., Newton A., Lim K.H. (2014). Clinical risk factors for preeclampsia in the 21st century. Obstet. Gynecol..

[32] Spradley F.T. (2017). Metabolic abnormalities and obesity’s impact on the risk for developing preeclampsia. Am. J. Physiol. Regul. Integr. Comp. Physiol..

[33] Lamminpää R., Vehviläinen-Julkunen K., Gissler M., Heinonen S. (2012). Preeclampsia complicated by advanced maternal age: A registry-based study on primiparous women in Finland 1997–2008. BMC Pregnancy Childbirth.

[34] Yang Y., Wu N. (2022). Gestational Diabetes Mellitus and Preeclampsia: Correlation and Influencing Factors. Front. Cardiovasc. Med..

[35] Spracklen C.N., Smith C.J., Saftlas A.F., Robinson J.G., Ryckman K.K. (2014). Maternal hyperlipidemia and the risk of preeclampsia: A meta-analysis. Am. J. Epidemiol..

[36] Alston M.C., Redman L.M., Sones J.L. (2022). An Overview of Obesity, Cholesterol, and Systemic Inflammation in Preeclampsia. Nutrients.

[37] Bartsch E., Medcalf K.E., Park A.L., Ray J.G., High Risk of Pre-eclampsia Identification Group (2016). Clinical risk factors for pre-eclampsia determined in early pregnancy: Systematic review and meta-analysis of large cohort studies. BMJ.

[38] Burton G.J., Jauniaux E. (2004). Placental Oxidative Stress: From Miscarriage to Preeclampsia. J. Soc. Gynecol. Investig..

[39] Moretti M., Phillips M., Abouzeid A., Cataneo R.N., Greenberg J. (2004). Increased breath markers of oxidative stress in normal pregnancy and in preeclampsia. Am. J. Obstet. Gynecol..

[40] Motedayen M., Rafiei M., Rezaei Tavirani M., Sayehmiri K., Dousti M. (2019). The relationship between body mass index and preeclampsia: A systematic review and meta-analysis. Int. J. Reprod. Biomed..

[41] Moslemi Zadeh N., Naghshvar F., Peyvandi S., Gheshlaghi P., Ehetshami S. (2012). PP13 and PAPP-A in the First and Second Trimesters: Predictive Factors for Preeclampsia?. ISRN Obstet. Gynecol..

[42] Goetzinger K.R., Singla A., Gerkowicz S., Dicke J.M., Gray D.L., Odibo A.O. (2010). Predicting the risk of pre-eclampsia between 11 and 13 weeks’ gestation by combining maternal characteristics and serum analytes, PAPP-A and free β-hCG. Prenat. Diagn..

[43] Hájek P., Macek M., Hladíková M., Houbová B., Alan D., Durdil V., Fiedler J., Malý M., Ostádal P., Veselka J. (2008). Pregnancy-associated plasma protein A and proform eosinophilic major basic protein in the detection of different types of coronary artery disease. Physiol. Res..

[44] Hantoushzadeh S., Ahangari R., Balaneji S.S., Ghamari A., Hashemnejad M., Piri S. (2023). Correlation of Fetal Heart Rate, Uterine Artery Pulsatility Index, Pregnancy Associated Plasma Protein-A and Crown-Rump Length in Pre-eclampsia—A Prospective Cohort Study. Maedica.

